# A quantitative review of the effects of Se application on the reduction of Hg concentration in plant: a meta-analysis

**DOI:** 10.3389/fpls.2023.1199721

**Published:** 2023-06-20

**Authors:** Jiefei Chen, Shangyan Hao, Gary Bañuelos, Xinbin Zhou

**Affiliations:** ^1^ College of Resources and Environment, Southwest University, Chongqing, China; ^2^ Institute of Soil Science, Chinese Academy of Sciences, Nanjing, China; ^3^ University of Chinese Academy of Sciences, Beijing, China; ^4^ United States Department of Agriculture-Agricultural Research Service, Parlier, CA, United States

**Keywords:** grains, mercury-selenium antagonism, meta-analysis, plant, rice

## Abstract

Mercury (Hg) is a highly toxic heavy metal entering the human body through the food chain after absorption by plant. Exogenous selenium (Se) has been suggested as a potential solution to reduce Hg concentration in plants. However, the literature does not provide a consistent picture of the performance of Se on the accumulation of Hg in plant. To obtain a more conclusive answer on the interactions of Se and Hg, 1,193 data records were collected from 38 publications for this meta-analysis, and we tested the effects of different factors on Hg accumulation by meta-subgroup analysis and meta-regression model. The results highlighted a significant dose-dependent effect of Se/Hg molar ratio on the reduction of Hg concentration in plants, and the optimum condition for inhibiting Hg accumulation in plants is at a Se/Hg ratio of 1–3. Exogenous Se significantly reduced Hg concentrations in the overall plant species, rice grains, and non-rice species by 24.22%, 25.26%, and 28.04%, respectively. Both Se(IV) and Se(VI) significantly reduced Hg accumulation in plants, but Se(VI) had a stronger inhibiting effect than Se(IV). Se significantly decreased the *BAF_Grain_* in rice, which indicated that other physiological processes in rice may be involved in restricting uptake from soil to rice grain. Therefore, Se can effectively reduce Hg accumulation in rice grain, which provides a strategy for effectively alleviating the transfer of Hg to the human body through the food chain.

## 1 Introduction

Mercury (Hg) is known to be a toxic trace element and enters the soil through a variety of ways (smelting, metal mining, coal burning, pesticides, fertilizers, sludge application, and sewage irrigation). It is taken up by plants and enters the food chain, eventually posing a serious threat to humans (Tan et al., 2018; Liu et al., 2019). The risk is greater for those who live on food sources grown in several contaminated regions (Feng et al., 2021).

Selenium (Se) is an essential trace element for human metabolism and normal physiological processes (Mao et al., 2016; Wang et al., 2018) and participates in various metabolic pathways to enhance immunity, regulation of thyroid function, anti-cancer properties, and even COVID-19 resistance (Ullah et al., 2019; Deng et al., 2021; Lima et al., 2021; Xia et al., 2021). However, approximately 51% of the soil in China is deficient in Se, and 70% of residents have an insufficient daily Se intake (Feng et al., 2013). Globally, approximately 0.5 to 1 billion people worldwide are considered to suffer from Se deficiency (Tan et al., 2016). Today, many serious medical complications, including Keshan disease, Kashin–Beck disease, cardiovascular diseases, poor immune function, cataracts, cognitive decline, and even cancer are related to Se deficiencies in the human body (Newman et al., 2019). Therefore, diets rich in Se is of practical importance to the population in Se-deficient regions (Liao et al., 2016). Crops and rice are a major Se source and food Hg exposure in diets (Zhang et al., 2012); therefore, we made rice the main object of study in this paper.

The application of Se fertilizer to plant not only can increase the Se content in crops and the Se intake in humans, but also reduces the Hg accumulation in crop plants (Li et al., 2015; Tang et al., 2017; Tran et al., 2018). The reduction of Hg accumulation in crops reduces Hg intake in the diet and protects human health. Fertilizing crops with Se with the aim to decrease Hg uptake and concentration in crops could be a win–win situation (Tran et al., 2018). Many researchers have studied the Se–Hg antagonism in soil–crop systems, although there are inconsistencies in their reported results. Some studies reported that the concentration of Hg in straw, brown rice tissues, and rice grains was significantly reduced (Tang et al., 2017; Tran et al., 2018). However, other studies showed that the accumulation of total Hg in the root, stem, leaf, and grains was enhanced with increasing Se(IV) and Se(VI) exposure dose (Zhao et al., 2020). So far, approximately 40 articles discussing the effect of Se application on Hg accumulation by plants have been published. However, large differences were observed between different experimental conditions, and even sometimes contradictory results. To gain a more conclusive answer on this Se and Hg interaction, a statistical evaluation was conducted by meta-analysis. We aimed to address the following: (1) How does exogenous Se application affect Hg uptake and accumulation by plant? (2) Do cultivation method, plant species, Se speciation, Hg speciation, Se/Hg molar ratio, growth stages, and accumulation organs differently influence Hg accumulation? (3) Do the present data support the proposed mechanisms reported in literature? We also highlight future studies between Se and Hg.

## 2 Materials and methods

### 2.1 Data sources

The data of this article come from the Web of Science [http://apps.webofknowledge.com (accessed on 1 February 2022)] and CNKI [https://www.cnki.net/ (accessed on 1 February 2022)]. Literatures in the database were retrieved using the following steps: (1) search the keyword “selenium or Se”; (2) search the keyword “mercury or Hg” from the results; (3) continue to search “plant or herb or woody plant or vegetable or grain” from the results; and (4) check the selected literatures according to the following literature screening criteria. The inclusion criteria were as follows: (1) literature marked with the species and cultivation methods of plant; (2) literature marked with the speciation of exogenous Se; and (3) literature marked with the speciation of determined Hg. After screening, we identified 38 literatures as the research scope (Notes S1).

In the process of extracting data from selected literatures, the results of treatments of the exogenous Se at different levels and species and other different extra treatments (e.g., different cultivation methods) in the same experiment were considered as independent measurements (De Graaff et al., 2006). Numerical data were directly extracted from the original document or table, and graphical data were extracted through Web Plot Digitizer 4.5 (https://apps.automeris.io/wpd/index.zh_CN.html), and the position error bar was assumed as the standard error (Treseder, 2004). A database was established using extracted data through Excel 2016, including the author, publication time, crop species, cultivation methods, application amount, and background concentration of Se and Hg, and the concentration units of Se and Hg were converted into mg·kg^−1^ L^−1^ and μmol·kg^−1^/L^−1^.

Rice (*Oryza sativa* L.) is not only a staple crop in the world, but also a major source of Hg and Se for humans (Jung et al., 2008; Ma et al., 2011; Filippini et al., 2018). Based on the overall plant data surveyed in this research, this paper focuses on the analysis of rice data. The data collected in this paper are divided into “Complete dataset”, “Rice only”, and “Rice excluded” (**Table 1**). By analyzing the collected data, this paper further studies the response of plant Hg accumulation to exogenous Se. Seven categorical variables are listed in **Table 2**, in which the forms of Hg in plant are divided into total Hg (THg), methylmercury (MeHg), and inorganic Hg (IHg). The Se/Hg molar ratio is the ratio of total Se (exogenous Se + background Se) concentration to total Hg (exogenous Hg + background Hg) concentration in growth media. The growth stage of rice is divided into seedling stage, elongation stage, booting stage, and maturity stage, while that of other plants is divided into seedling stage and maturity stage. The plant Hg BAF (bio-accumulation factor) is divided into *BAF_Root_*, *BAF_Stem_*, *BAF_Leaf_*, and *BAF_Grain_*, which respectively represent the bio-accumulation factors in different plant tissues.

**Table 1:** Heterogeneity test results of datasets.

Index	Total dataset	Rice only	Rice excluded
*n*	1,193	517	676
*Q*_T_	1,558,008.11^***^	1,329,861.48^***^	69,354.97^***^
*I* ^2^(%)	99.92	99.96	99.02

Number of observations (*n*); total heterogeneity (*Q*_T_); percentage of heterogeneity (*I* ^2^). Significance of the *Q*_T_: ns; *; **; *** = not significant; significant at *p*< 0.05; significant at *p*< 0.01; significant at *p*< 0.001, respectively.

**Table 2:** Categorical variable level and heterogeneity test results.

Q_B_ (Categorical variable)	L1	L2	L3	L4	L5	Total dataset	Rice only	Rice excluded
*Q*_B_ (Cultivation method)	Pot	Hydroponic	Sand	Field	Foliar	18.73^***^	3.75^***^	94.69^***^
*Q*_B_ (Exogenous Se speciation)	Se(IV)	Se(VI)	–	–	–	0.37^ns^	0.49^ns^	0.005^ns^
*Q*_B_ (Plant Hg speciation)	THg	MeHg	IHg	–	–	4.77^**^	1.30^*^	–
*Q*_B_ (Se/Hg molar ratio)	≤1	1–3	>3	–	–	10.26^**^	9.39^**^	34.66^***^
*Q*_B_ (Plant Hg BAF)	Root	Stem	Leaf	Grain	–	202.63^***^	100.50^***^	28.74^***^
*Q*_B_ (Growth stage)	Seedling	Elongation	Booting	Mature	–	2.10^***^	1.72^*^	3.42^ns^
*Q*_B_ (Plant part)	Root	Stem	Leaf	Grain	–	–	1.56^*^	–

Specific levels (L); between-group heterogeneity (*Q*_B_); no content (−). Significance of the *Q*_B_: ns; *; **; *** = not significant; significant at *p*< 0.05; significant at *p*< 0.01; significant at *p* < 0.001, respectively.

The *BAF* was calculated as follows:


(1) $$BAF_{tissue}=C_{tissue}/C_{growth\text{ media}}$$

where *C*_tissue_ and *C*_growth media_represent the Hg concentration of plant tissue and growth media, respectively. All units are μmol·kg^−1^. A higher *BAF* represented a stronger absorption capacity of Hg.

### 2.2 Statistical analysis

We used the method of natural log-transformed response ratio (ln*R*) (Hedges et al., 1999) and MetaWin 2.1 software to calculate the effect size and variance for each pair of data. The formulas are as follows:


(2) $$InR=(X_{E}/X_{C})$$

(3) $$v\text{ = }\frac{SD_{E}^{2}}{n_{E}X_{E}^{2}}+\frac{SD_{C}^{2}}{n_{C}X_{C}^{2}}$$

where ln*R* is the natural log-transformed response ratio and is defined as the effect size; *X_E_* and *X_C_* are the average Hg content of plant in the experimental treatment (exogenous Se) and control treatment, respectively; *n_E_* and *n_C_* are the replicate numbers; *SD_E_* and *SD_C_* are the standard deviations. When no standard error, standard deviation, or confidence interval (CI) is provided, we assumed that the standard deviation is 1/10 of the mean (Xiang et al., 2017).

**Figure 1:**
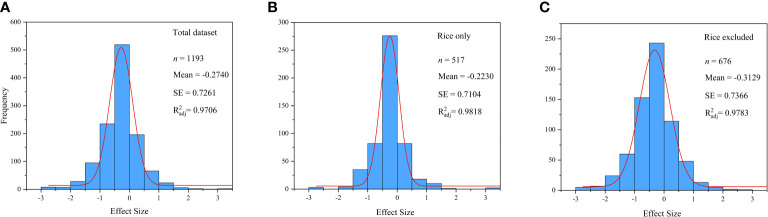
Frequency distribution of the ln*R*. **(A)** Total dataset. **(B)** Rice only. **(C)** Rice excluded.

The weighted mean effect size was calculated by weighted analysis. Before the weighted analysis, the frequency distributions of ln*R* (**Figure 1**) and the Gaussian function fitting curve were plotted by the ORIGIN PRO 2021 to verify the normality of the data (Ainsworth, 2008). In the weighted analysis, a weighted random-effects model was used to calculate the overall effect size, grouped effect size, and 95% confidence interval (95% CIs) (Dieleman et al., 2010). The response to Hg accumulation in plant was considered significant (*p*< 0.05), if the 95% CIs did not overlap with 0 (Hedges and Olkin, 1985). The percentage change in response is calculated as (*R −* 1) × 100% (De Graaff et al., 2006). A negative value for the percentage change in response means that Se addition had a negative effect on Hg concentration, while positive values indicate a beneficial effect.

The effect of taxonomic variables on Hg accumulation in plant was studied by subgroup analysis. Before the subgroup analysis, total heterogeneity (*Q*_T_) was used to quantify the overall degree of difference of the effect sizes between different paired observations in the same variable, following the rule of *k −* 1 degrees of freedom *χ* ^2^ distribution [*k* is the number of paired observations between experimental treatment (exogenous Se) and control treatment] (Rosenberg et al., 2000). Heterogeneity was indicated when *p*< 0.05 was associated with *Q*_T_. Heterogeneity, which was also reassessed using the *I* ^2^ index, quantified the ratio of real variation to total variation (real variation + sampling error) caused by actual differences between studies (**Table 1**) (Huedo-Medina et al., 2006). When the *I* ^2^ value is positive, this also indicates heterogeneity (Wang et al., 2017). In the subgroup analysis, the total heterogeneity (*Q*_T_) of each categorical variable was divided into within-group heterogeneity (*Q*_w_) and between-group heterogeneity (*Q*_B_) (Hu et al., 2021). If the *Q*_B_ of the categorical variable is significant (*p*< 0.05), the variable is subdivided into different levels (**Table 2**) (Wittig et al., 2009), and the forest graphs were drawn by GraphPad Prism 8. If there is at least 10 observations or observations from at least five independent articles in a level, the level will be included in the analysis; otherwise, it will be excluded in the analysis (Feng et al., 2010). If the 95% CIs of the two levels did not overlap, a significant difference was considered between them at α = 0.05 (Gurevitch and Hedges, 1999). Otherwise, the *t*-test was used to further test whether the difference between the two level is significant (Liao et al., 2008).

**Figure 2:**
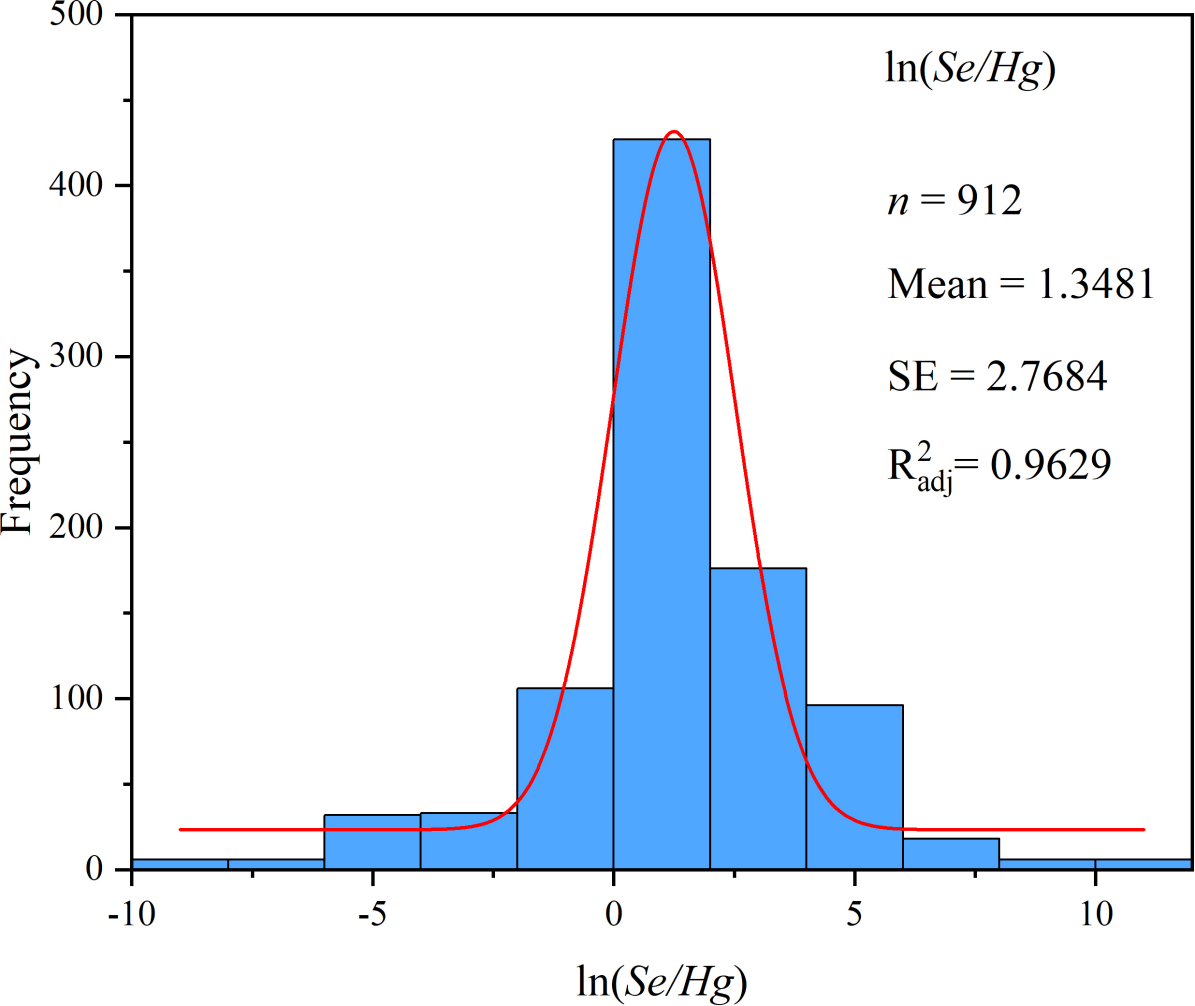
Frequency distribution of the ln(*Se/Hg*).

The effect of continuous variables on Hg accumulation in plant was studied by meta-regression analysis. The continuous meta-analysis model was used to analyze the relationship between the effect size (ln*R*) and the continuous variable (Se/Hg). When the slope was not 0 and *p*< 0.05, the regression relationship was considered to be significant (Rosenberg et al., 2000; Zhou et al., 2014), and the regression result graphs were drawn by ORIGIN PRO 2021. Because the value range of exogenous Se concentration was too large, the Se/Hg value (μmol·kg^−1^/L^−1^) was converted into natural logarithm ln(*Se/Hg*) (Affholder et al., 2019), and the normality test results are shown in **Figure 2**.

In the process of confirming publication bias, Kendall’s tau rank correlation and Spearman’s Rho rank correlation between the standardized effect size and variance of each observation were used to statistically access the possibility of publication bias of each categorical variable and each level (Rosenberg et al., 2000). Afterwards, a Rosenthal fail safe number at α = 0.05 was calculated (Rosenthal, 1979). Publication bias can be confirmed only when the results of the above two rank correlation tests were significant (*p*< 0.05) and the Rosenthal failure safety number was less than 5*k* + 10 (where *k* is the observation number) (Hu et al., 2021).

## 3 Results

### 3.1 Overall dataset

**Table S1** lists a detailed overview of the 38 articles collected in this paper, including 16 crops and 1,193 pairs of valid data. Exogenous Se had a concentration of 0.001–6,332.32 μmol·kg^−1^/L^−1^, while total Se concentration in growth media (exogenous Se + background Se) is in the range of 0.01–6,335.74 μmol·kg^−1^/L^−1^. The total Hg concentration in growth media (exogenous Hg + background Hg) was in the range of 0–1,179.67 μmol·kg^−1^/L^−1^. Se/Hg molar ratio was in the range of 0.00–25,404.00, with an average of 310.22. The average reduction of plant Hg accumulation was 12.05 ± 155.92 μmol·kg^−1^. The effect of exogenous Se on Hg accumulation in plant was investigated based on pot, hydroponic, sand, field, and foliar fertilization experiments, with the data accounting for 48.11% (18 papers), 26.82% (13 papers), 13.08% (7 papers), 8.30% (4 papers), and 3.69% (3 papers), respectively (**Table S2**).

**Figure 3:**
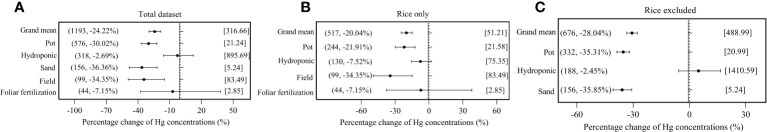
Effects of exogenous Se on Hg accumulation in plant under different cultivation methods. **(A)** Total dataset. **(B)** Rice only. **(C)** Rice excluded. The size of the abscissa corresponding to the dot represents its pooled effect size, and the error bar represents 95%. The data in brackets respectively represent the number of observations and the size of the pooled effect size corresponding to the dots.

As illustrated in **Figure 3**, there were significant (*p*< 0.05) responses of overall plant species on Hg accumulation to exogenous Se (−24.22%), on rice Hg accumulation to exogenous Se (−20.04%), and in non-rice species on Hg accumulation to exogenous Se (−28.04%). In reference to cultivation method, pot culture, sand culture, and field experiments, all showed a significant impact on the interaction of Se and Hg in the three data groups, while hydroponic culture and foliar fertilization experiments showed no significant impact in the three data groups (*p* > 0.05).

### 3.2 Exogenous Se speciation

**Figure 4:**
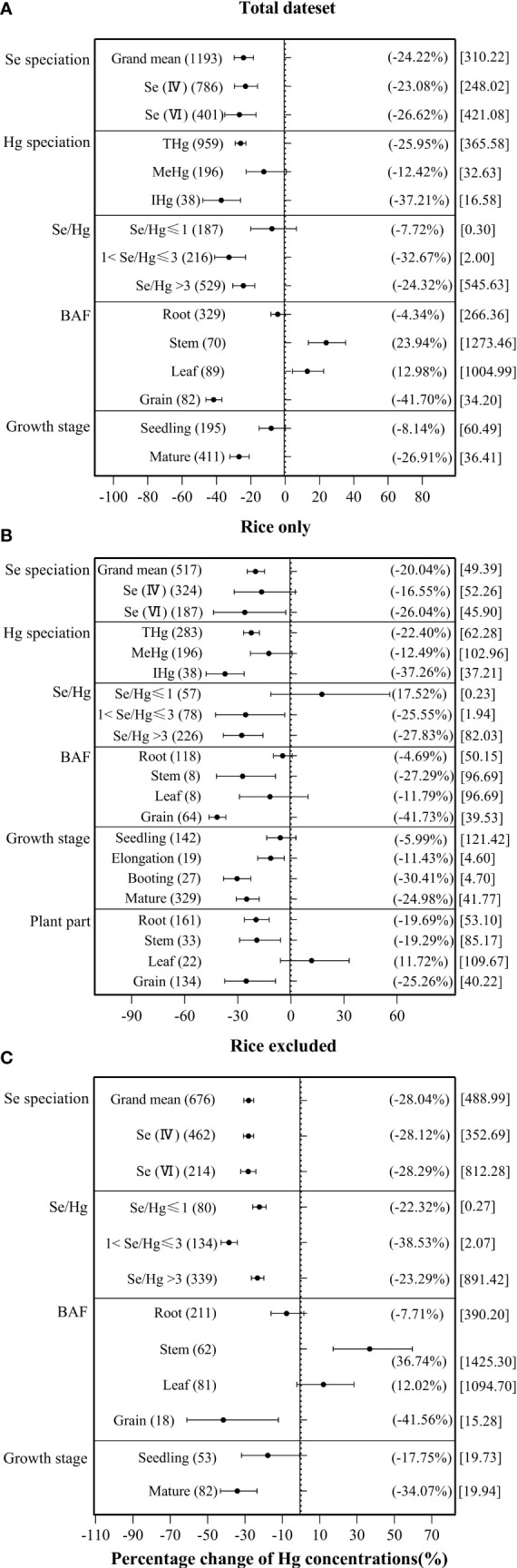
The effects of exogenous Se on the Hg accumulation in plant at different levels of categorical variables. **(A)** Total dataset. **(B)** Rice only. **(C)** Rice excluded. The size of the abscissa corresponding to the dot represents the size of its combined effect value, and the error bar represents 95%. The value in the bracket on the left side of the figure indicates the number of observed values; the value in the right bracket indicates the size of the combined effect value corresponding to the dot, and the value in the square bracket indicates the Se/Hg molar ratio corresponding to the level of the categorical variable.

According to **Figure 4A**, both exogenous Se(IV) and exogenous Se(VI) significantly inhibited plant Hg accumulation by −23.08% and −26.62%, respectively. According to **Figure 4B**, exogenous Se(VI) has a significant inhibitory effect (−26.04%) on Hg accumulation in rice, while exogenous Se(IV) has no significant inhibitory effect (−16.55%) on Hg accumulation in rice. According to **Figure 4C**, both exogenous Se(IV) and exogenous Se(VI) significantly inhibited by −28.12% and −28.29% Hg accumulation, respectively, in non-rice species. ANOVA showed that there was no significant difference between exogenous Se(IV) and Se(VI) in inhibiting Hg accumulation in plant.

### 3.3 Plant Hg speciation

For different Hg forms in plant, the inhibition rate of exogenous Se against the THg accumulation in plant was 25.95%, compared to 12.42% in MeHg, and to 37.21% in IHg, indicating that exogenous Se has significantly greater inhibitory effect on IHg accumulation in plant than on MeHg accumulation (**Figure 4A**). In rice, exogenous Se also has significantly greater inhibitory effect on rice IHg (−37.26%) than on MeHg (−12.49%) (**Figure 4B**).

### 3.4 Se/Hg molar ratio

Because the experimental background of each group of data is different, the index of Se/Hg molar ratio is used in this paper. For the overall plant species, when 1< Se/Hg ≤ 3 (−32.67%), Se/Hg > 3 (−24.32%), Hg accumulation was significantly reduced (**Figure 4A**). For rice, when 1< Se/Hg ≤ 3 (−25.55%), Se/Hg > 3 (−27.83%), Hg accumulation was significantly reduced (**Figure 4B**). For non-rice species, when Se/Hg ≤ 1 (−22.32%), 1< Se/Hg ≤ 3 (−38.53%), Se/Hg > 3 (−23.29%), Hg accumulation was also significantly lower (**Figure 4C**).

**Figure 5:**
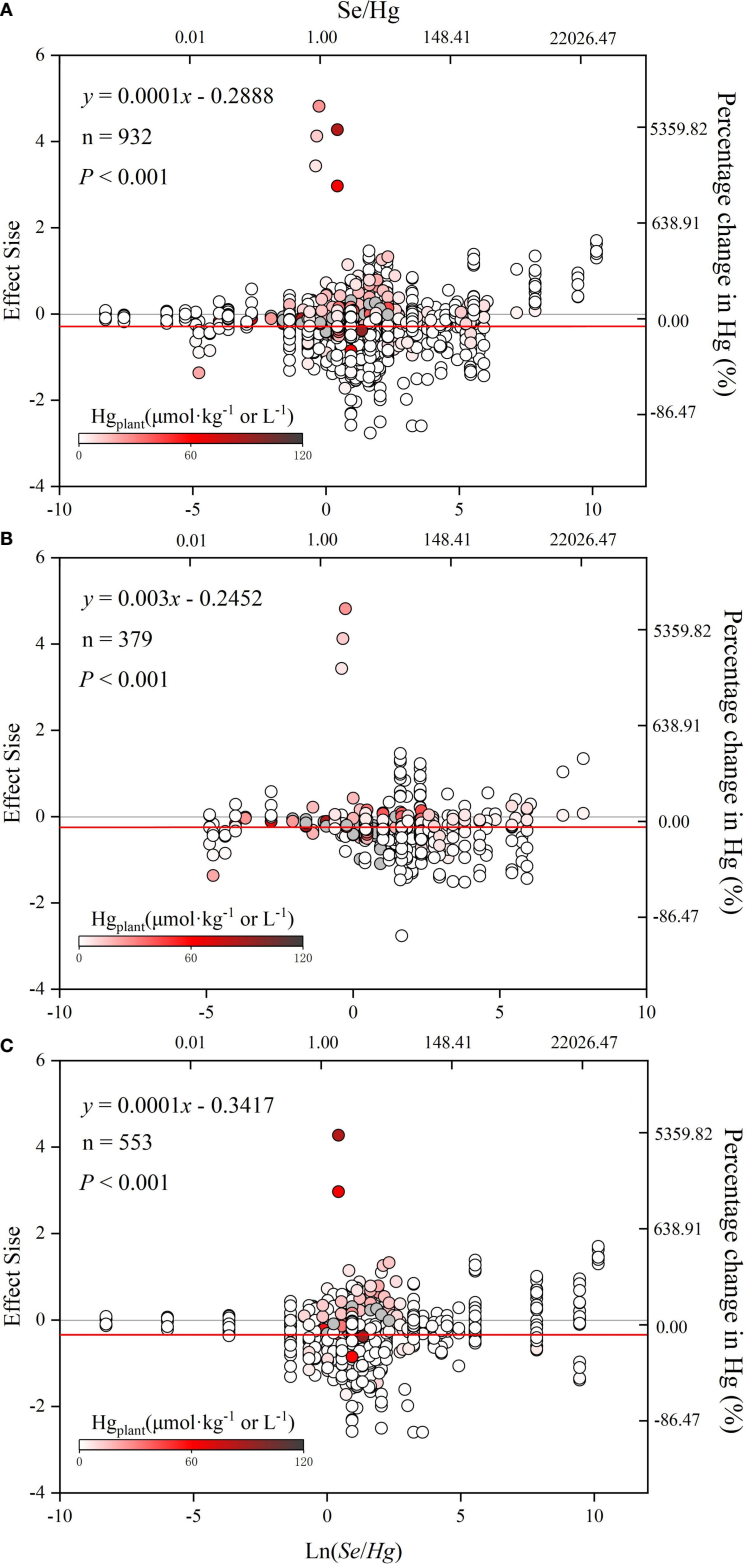
The relationship between effect size (ln*R*) and ln(*Se/Hg*). The graph of meta regression between the continuous variable [Se/Hg value (logarithmic conversion), lower horizontal axis] and the effect size of the response variable. **(A)** Total dataset. **(B)** Rice only. **(C)** Rice excluded. The solid line represents the results of meta regression, and the dotted line represents the 95% CIs.

According to **Figure 5**, ln*R* is significantly positively correlated with ln(*Se/Hg*) (*p*< 0.05), indicating that the reduction of Hg accumulation in plant depends on the size of Se/Hg. Results of the meta-analysis performed on the whole dataset and non-rice dataset (**Figures 5A–C**) indicated a significant dose-dependent effect of Se/Hg molar ratio on reduction of Hg concentration in plants.

### 3.5 Plant Hg BAF

Some studies reported that the majority of IHg in rice leaf is derived from ambient air. Thus, BAF is selected to reflect the bioaccumulation of Hg in different tissues (Meng et al., 2012). For plant Hg BAF, the inhibition rate of exogenous Se on overall plant *BAF_Root_* and *BAF_Grain_* are 4.34% and 41.70% respectively, while the promotion rate on overall plant *BAF_Stem_* and *BAF_Leaf_* is 23.94% and 12.98%, respectively (**Figure 4A**). The inhibition rates of rice *BAF_Stem_* and *BAF_Grain_* are 4.69% and 41.73% significantly, but *BAF_Root_* and *BAF_Leaf_* are 4.69% and 11.79% non-significantly (**Figure 4B**). The inhibition rate to non-rice species *BAF_Root_* and *BAF_Grain_* is 7.71% and 41.56%, respectively, while the promotion rate to other plant *BAF_Stem_* and *BAF_Leaf_* was 36.74% and 12.02%, respectively (**Figure 4C**). It shows that exogenous Se has a more significant inhibitory effect on *BAF_Root_* and *BAF_Grain_*, and the inhibitory effect on *BAF_Grain_* is greater than that on *BAF_Root_*.

### 3.6 Plant growth stage

In terms of plant growth stage, the Hg accumulation with exogenous Se at the seedling stage decreased by 8.14%, and by 26.91% at the mature stage, indicating that the inhibition of exogenous Se on Hg at the mature stage was significantly greater than that in the seedling stage (**Figure 4A**). In rice, exogenous Se has the strongest inhibition on Hg accumulation at the booting stage (−30.41%) (**Figure 4B**). For non-rice species, exogenous Se reduced Hg accumulation by 17.75% at the seedling stage and 34.07% at the mature stage.

### 3.7 Publication bias

The normal distribution diagram (**Figure 1**) shows that the data used in this paper obey the normal distribution Radj 2 ( 0.9), which reveals that there is no publication bias in this paper to a certain extent. In addition, **Table 3** lists the calculation results of publication bias of the results with significant CIs in subgroup analysis, which further reveals that there is no publication bias in this paper.

**Table 3:** Kendall’s tau and Spearman’s rho rank correlation tests, as well as Rosenthal’s fail-safe numbers for assessing publication bias.

Variable	Kendall’s tau	Spearman’s rho	Rosenthal’s fail-safe numbers
Complete dataset	−0.057^**^	−0.08^**^	138,529,700.3
Rice only	0.011^ns^	0.015^ns^	49,955.1
Rice excluded	−0.128^**^	−0.162^**^	143,869,145.2

No publication bias if the *p*-values of Kendall’s tau and Spearman’s rho rank correlation tests are > 0.05, and the Rosenthal’s fail-safe numbers are greater than 5*k* + 10, where *k* is the observation number.

ns, not significant; **, significant at p< 0.01.

## 4 Discussion

### 4.1 Values of the Se/Hg molar ratio affecting Hg accumulation in plant

**Figure 6:**
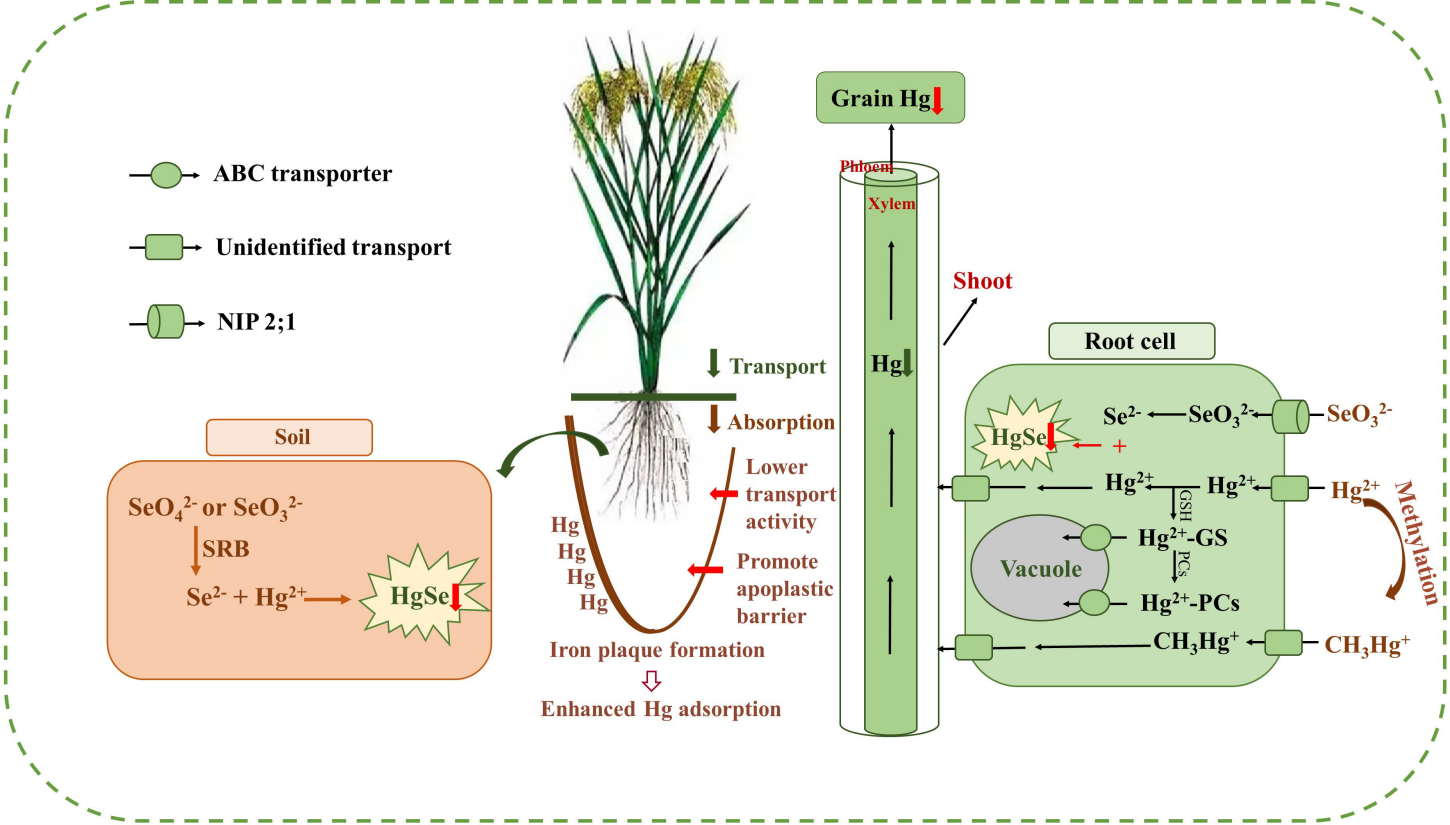
The outline of the mechanisms of Se reducing Hg accumulation in rice. Exogenous Se can help to form the insoluble HgSe in the soil and roots, which are restricted in their upward Hg migration (red down arrow). Meanwhile, exogenous Se can enhance Hg adsorption by promoting iron plaque formation and inhibit the transportation of Hg from roots to shoots and grains (green down arrow) and reduce the absorption of Hg in roots (brown down arrow) by inducing the decease of membrane transporter activity and promoting the formation of apoplastic barriers in the root endoderm (red right arrow). Afterwards, exogenous Se can also promote the sequestration of Hg in vacuoles.

Results of the meta-analysis performed on the overall dataset, rice-only dataset, and rice-excluded dataset indicated a significant, dose-dependent effect of the Se/Hg molar ratio on reduction of Hg concentration in plant (**Figures 5A–C**). Further meta-subgroup analysis showed that the antagonistic effects of Se on Hg accumulation in plant depended on the Se/Hg molar ratio in growth media (**Figures 4** and **5**). When Se/Hg ≤ 1, Se cannot significantly reduce Hg accumulation in plant. However, when 1< Se/Hg ≤ 3, Se significantly reduced Hg concentrations in plant, and its inhibition on Hg was stronger than that of Se/Hg > 3, which is to say that the optimum condition for inhibiting Hg accumulation in plant is at a Se/Hg molar ratio of 1–3. Although the interaction effect of Se and Hg in crops was shown, the mechanisms involved are still not well understood; we outline potential mechanisms found in the literature (**Figure 6**) that could participate in the interaction of Se and Hg in soil and crop in the discussion below.

Our results were consistent with the previous research results. For example, Zhao et al. (2013) found that when the growth media had low Hg content (0.01–1 mg·L^−1^), the Hg content of garlic increased with increasing concentration of $SeO_{3}^{2-}$ and $SeO_{4}^{2-}$ (1–100 mg·L^−1^), but phytotoxicity symptoms were likely caused by excess Se in growth media. In addition, the different effects of exogenous Se on Hg accumulation in plant may be related to the reason for the formation of nonbioavailable Hg–Se complexes on the root surface and in the soil when the Se/Hg molar ratio is 1–3 or higher (McNear et al., 2012). To sum it up, an effective inhibition of Hg accumulation in plant required that the Se/Hg molar ratio be within a certain range in the growth media.

In agricultural production, Hg pollution is becoming more and more serious. For this reason, Se fertilizer application is a cost-effective and win–win strategy to reduce Hg concentrations, promote agro-environmental sustainability and food safety, and decrease the public health risk posed by Hg-contaminated soils and its accumulation in food crops (Tran et al., 2021). The results of this study provide a reference for the use of Se as a strategy to reduce Hg accumulation in food production. The Chinese national standard GB 15618-2018 *Soil Environmental Quality - Standard for Soil Pollution Risk Control on Agricultural Land (Trial)* reports that soils with a mercury content greater than 2.5 mg·kg^−1^ (5.5< pH ≤ 6.5) may have a soil pollution risk (GB/T 15618-2018, 2018). According to the conclusion in this article that the best condition for inhibiting the accumulation of Hg in plants is a Se/Hg molar ratio of 1–3, it is known that when the soil selenium content is 0.98–2.95 mg·kg^−1^, the accumulation of Hg in plants can be significantly reduced. In Hg mining areas, Hg and Se always co-exist in the soil, such as the Wanshan mining area, Guizhou, China (Zhang et al., 2022). Se concentrations were positively correlated with concentrations of both IHg and MeHg in the soils (Liu et al., 2019). If Se/Hg ≤ 1 in Hg-contaminated soil, a low-Hg and Se-rich crop can be produced by just supplying a small amount of Se fertilizer to the soil. This inexpensive strategy is an effective use of Se fertilizer in agricultural production to reduce Hg accumulation in crops.

### 4.2 Factors of exogenous Se reducing Hg accumulation in plant

Exogenous Se reduced the Hg accumulation of the overall plant species, rice, and non-rice species by 24.22%, 20.04%, and 28.04%, respectively (**Figure 3**). In this paper, the effect of exogenous Se on plant Hg accumulation depended on the exogenous Se speciation, plant Hg speciation, Se/Hg ratio, plant Hg BAF, and plant growth stage of applying Se to reduce Hg, which were further explored.

Meta-analysis showed that both Se(VI) and Se(IV) can significantly reduce Hg concentration in plants (**Figure 4**), which is consistent with most research results (Wang et al., 2016a; Wang et al., 2016b; Wang et al., 2016c; Tang et al., 2017). In addition, meta-analysis also showed that Se(VI) application can reduce the concentrations of Hg more than Se(IV) in plants (**Figure 4**). Tang et al. (2017) also showed significant differences in the inhibitory effect of different forms of exogenous Se on the accumulation of Hg^2+^ in rice. Wang et al. (2016a) pointed out that $SeO_{3}^{2-}$ has a greater inhibitory effect than $SeO_{4}^{2-}$ , which is contrary to the results of our study. In this regard, the different phenomena can be ascribed to the differences in Hg among various plant species and different experimental conditions. However, the results in this study are based on a comprehensive analysis of different plant species and different experimental conditions, which can help identify selenium modified species that can be used for stabilizing mercury plants in future research.

Nanomaterials can be used as new slow-release fertilizers to improve nutrient utilization, not only reducing costs but also protecting the environment (Elshahawy et al., 2018). The exploration of biosynthesized nanoparticle-based fertilizers and nano-biofertilizers is a promising and outstanding technology for increasing and optimizing productivity (Ali et al., 2021). For example, Hussein et al. (2019b) found that nano-selenium (Nano-Se), acting as a stimulator and/or stressor, enhanced the antioxidant defense system of tested groundnut cultivars, thus improving the tolerance of plants under sandy soil conditions. Hussein et al. (2019a) also found that the tested Nano-Se concentrations increased peanut yield and seed oil content. Moreover, a study has found that bacterial selenium nanoparticle (Se-NP) complexes may be used as bioremediation tools for pathogens in required water (El-Shanshoury et al., 2020). Compared with $SeO_{3}^{2-}$ and $SeO^{4-}$ , the naturally occurring Se-NPs produced by the reduction of $SeO_{3}^{2-}$ through non-biological and biological processes have lower phytotoxicity and stronger capacity for Hg sequestration in garlic (Zhao et al., 2020). For instance, Zhu et al. (2022) found that biosynthetic Nano-Se significantly reduced the Hg concentration in roots and shoots of pak choi. However, Xie et al. (2023) found that nano-sized HgSe (Nano-HgSe) can be decomposed and can serve as an environmental source of Hg in rice. Therefore, more work is needed to investigate the factors that influence Nano-HgSe formation and the mechanism of Hg methylation after exposure of rice to Nano-HgSe.

The present meta-analysis showed that exogenous Se can significantly reduce THg and IHg content in plants. THg accumulation by plant was more inhibited by exogenous Se than MeHg accumulation. The above analysis is consistent with the previous research results. MeHg was more concentrated in rice grains than roots, stems, and leaves, while IHg was highest in roots, indicating that MeHg was more efficiently absorbed and loaded in grains than IHg, and Se showed much stronger inhibition on IHg compared with MeHg (Zhang et al., 2010; Rothenberg et al., 2011; Rothenberg et al., 2014). Zhao et al. (2014) demonstrated that the content of IHg in roots, stalks, leaves, and grains of rice was decreased with Se supplement, and IHg content had significantly greater reduction than MeHg. This observation can be attributed to the direct inhibitory effect of Se on IHg, while the inhibitory effect on MeHg is weakly produced indirectly by significantly reducing Hg^2+^ concentration and suppressing Hg methylation (Tran et al., 2021). Exogenous Se can also promote the synthesis of glutathione (GSH) and phytochelatin (PC) in plant tissues. Hg^2+^ chelated with GSH and PC in the cytoplasm of root cells forms HG–PCs or HG–GS complexes (McNear et al., 2012; Han et al., 2015). ATP-binding cassette (ABC) transporters transport the complexes into vacuoles and sequestered them, which inhibits the transfer of Hg^2+^ from rice roots to shoots (Park et al., 2012; Sharma et al., 2016). However, there were no CH_3_Hg^+^–PCs complexes, suggesting that exogenous Se had a stronger inhibitory effect on IHg in plant, but not for CH_3_Hg^+^ (Krupp et al., 2009).

Analysis of plant growth stage reveals that exogenous Se can inhibit Hg accumulation in plants more at the mature stage than at the seedling stage and it has the strongest inhibitory effect on Hg accumulation at the booting stage of rice, which indicates that the key stage to determine Hg content in rice is probably from the booting stage to the mature stage. Zhang et al. (2017) also found that the size of the primary transfer coefficient (PT1) in each growth stage of rice is booting stage > maturity stage > jointing stage, which is probably because the Se content is high in the stems and leaves of rice at maturity stage. At this stage, the application of Se fertilizer will activate the physiological mechanism of rice itself to resist Se toxicity by improving the absorption and enrichment of Hg, a synergistic effect of Se and Hg (Zhao et al., 2013). Based on this conclusion, it is essential for farmers to be aware of growing plants with Se fertilization at the booting stage to remediate the Hg accumulation, and to pay attention to the Se/Hg ratio in the agricultural soil before supplying Se fertilization. This precaution will also reduce the cost of Se application.

Finally, the geochemical factors that interact with Se and Hg are very complex. Other geochemical factors need to be studied, such as soil type, soil moisture, and different pH conditions.

### 4.3 Mechanisms of exogenous Se reducing Hg accumulation in rice

Se significantly decreased the *BAF_Grain_* in rice (**Figure 4**), which indicated that physiological processes in rice may be involved in restricting uptake from soil to rice. Zhao et al. (2020) also reported that the micro-distribution patterns of Hg mainly concentrated in the root surface and root rather than in the leaf after Hg and Se co-exposed. In addition, foliar application of Se also has no effect of Hg accumulation in rice grains and grape berries (*Vitis vinifera* L.) (Zhu et al., 2017). This observation is an example that shows how Se restricts Hg accumulation in plants’ roots (**Figures 3** and **4**). The main mechanism of how exogenous Se reduces Hg accumulation in rice will be discussed below (**Figure 6**).

From the perspective of soil, under anoxia conditions, exogenous Se will go through a series of transformation processes and eventually form HgSe precipitates with Hg^2+^ to reduce the effective Hg^2+^ content, and then inhibit the accumulation of Hg in rice. Tang et al. (2017) also reported that soil Se application inhibited the absorption Hg^2+^ by rice, which can be attributed to the fact that exogenous Se can be transformed into $SeO_{3}^{2-}$ or $SeO_{4}^{2-}$ under anoxic or suboxic conditions and can react thermochemically with Hg^2+^ to form HgSe complexes. Soil CH_3_Hg^+^ concentrations were consistently lower after Se treatments, which may be predominantly governed by sulfate-reducing bacteria (SRB) under anoxic and suboxic conditions (Tran et al., 2021). Zhang et al. (2012) indicated that the absorption of Hg in rice tissues from the rice root to the aerial part was significantly reduced, which might be due to the reduction of free $SeO_{3}^{2-}$ or $SeO_{4}^{2-}$ into Se^2−^ by SRB (Li et al., 2014). Se^2−^ can combine with free Hg^2+^ to form HgSe complexes and reduce bioavailable Hg^2+^ content in rhizosphere soil. Thus, the absorption of Hg from soil by plant roots was inhibited. TEM-EDX analysis also confirmed that the molar ratio of Hg to Se was approximately 1:1 in HgSe nanoparticles in Se-amended soils, providing strong evidence for the formation of HgSe complexes in soil (Wang et al., 2016a). In addition, Wang et al. (2019) indicated that the decrease in grain MeHg levels after the combination of Se and biochar amendments was partly attributed to the inhibition of Se on the net yield of MeHg in soil. The above analysis indicated that exogenous Se can reduce the accumulation of Hg in rice by decreasing the bioavailability of Hg in soil.

From the perspective of soil–rice root interface, exogenous Se can reduce the absorption of Hg in rice by promoting the formation of iron plaque on root surface, decreasing the activity of membrane transporters, and promoting the formation of apoplastic barriers in the root endoderm (Meyer et al., 2009; Ding et al., 2014; Wang et al., 2014; Li et al., 2015; Zhang et al., 2017). Exogenous Se can activate manganese and iron in soil and promote the formation of iron plaque on root surface, making it a natural barrier for plant roots to absorb Hg. Specifically, Se^2−^ exhibited a strong reducing ability, reducing the high valence of Fe and Mn in the soil, thereby increasing the concentration of Fe^2+^ and Mn^2+^ in the soil solution (Huang et al., 2019). The iron plaque sequesters Hg through adsorption or co-precipitation, reduces the bioavailable Hg content in the rhizosphere, and suppresses the absorption of Hg^2+^ and $CH_{3}Hg^{+}$ in the roots (Zhou and Li, 2019; Huang et al., 2020). Exogenous Se can also promote the formation of apoplastic barriers in the root endodermis, restricting the absorption of Hg through the apoplastic pathway (Wang et al., 2014). The above analysis indicated that exogenous Se can reduce the accumulation of Hg in rice by inhibiting the absorption of Hg in roots.

From the perspective of rice roots, exogenous Se not only can form insoluble HgSe with Hg in rice root, but also can promote the sequestration of Hg in the vacuole of rice root cells. Exogenous Se can transform the unstable Hg into insoluble HgSe in the root, thus inhibiting the transfer of Hg from the root to the shoot of rice (Zhang et al., 2012). The field test results of Li et al. (2015) also showed that a certain concentration of exogenous Se can significantly reduce the concentration of Hg in rice grains, and synchrotron radiation x-ray fluorescence (SRXRF) technique revealed that HgSe polymers were found in rice roots (Zhao et al., 2014), which was unavailable for plant uptake. The upward translocation of Hg through the root vessel to the leaf may be obstructed after Se application. Moreover, Krupp et al. (2009) identified that Hg^2+^–PC, but no $CH_{3}Hg^{+}–PC$ complexed in the rice roots, suggesting that the binding to PC may inhibit the translocation of Hg^2+^ from rice roots to stems, but not $CH_{3}Hg^{+}$ (**Figure 4A**). In addition, Se amendment can enhance the development of apoplastic barriers in the root endodermis and exodermis, which can reduce the uptake of Hg by roots (Wang et al., 2014). The above analysis indicated that exogenous Se can sequester Hg in rice roots.

The antagonism of Se–Hg from soil to root is a key process controlling Hg accumulation in rice grains (**Figure 4**). With foliar Se application, grain IHg did not decrease significantly compared with soil Se application (**Figures 3A, B**), suggesting that IHg–Se antagonism mainly occurs in the soil and roots. This is consistent with previous research results (Tang et al., 2017). Stable Hg isotope techniques showed that approximately 20% of Hg in grains was from the atmosphere, which also suggests that the soil is the dominant IHg source for the rice grain (Feng et al., 2016). Therefore, Se plays an important role in reducing the bioavailability of Hg in the soil plant system, but the mechanism has not yet been fully elucidated.

It is necessary to deeply study the mechanism of Se to reduce Hg accumulation before and after it enters plants, and the mechanism of Se to prevent Hg entry into the grain. This understanding is of practical importance for effectively reducing the risk of human exposure to Hg and solving the problem of insufficient dietary Se intake.

## 5 Conclusions

In this paper, 1,193 data records were collected from 38 publications for this meta-analysis. We tested the effects of different factors on Hg accumulation by meta-subgroup analysis and meta-regression model. The results highlighted a significant dose-dependent effect of the Se/Hg molar ratio on reduction of Hg concentration in plants and identified the optimum conditions for inhibiting Hg accumulation in plants with a Se/Hg ratio of 1–3. Exogenous Se significantly reduced Hg concentrations of the overall plant species, rice grains, and non-rice species by 24.22%, 25.26%, and 28.04%, respectively. Both Se(IV) and Se(VI) significantly reduced Hg accumulation in plants, but Se(VI) had a stronger inhibiting effect than Se(IV). Se significantly decreased the *BAF*_Grain_ in rice, which indicated that other physiological processes in rice may be involved in restricting uptake from soil to rice grain. Therefore, Se can effectively reduce Hg accumulation in rice grain, which provides a potential management strategy for producing low-Hg and Se-rich crops, and thereby reducing the Hg intake from human diet.

We should continue to study the effects of Se on Hg accumulation in plant from the following aspects:

1. To reduce human intake of Hg, we suggest to further explore the antagonistic mechanisms between different forms of Se and Hg in the soil–plant system. For example, it is recommended to conduct more research on the impact of Nano-Se on plant Hg accumulation in the future.
2. To clarify the best Se application strategy, we suggest to further explore the possibility of mixed strategies to reduce Hg accumulation by combining Se with other strategies (water management, biochar, and other beneficial schemes) through field experiments. The concentration range of Se from beneficial to harmful effects is very narrow. Reduce the Hg accumulation in rice to improve the Se level and ensure that the Se content does not exceed the standard. More research should be done to determine the safe range of Se use in edible plants.
3. Forms of IHg and MeHg in rice grains have been proven to primarily originate from soil, which provides ideas for potential *in situ* remediation of Hg-contaminated soil. Before applying Se amendments in Hg-polluted contaminated paddies, it is necessary to study the dynamic balance of Hg after applying Se. Other factors need to be considered when applying Se fertilizer, such as the most suitable soil type and soil humidity, pH, organic content, and redox potential.

## Author contributions

JC: Data curation, Software, Writing- Original draft preparation, Visualization. SH: Data curation. GB: Writing-Review and Editing. XZ: Conceptualization, Supervision, Writing-Original draft preparation, Writing-Review and Editing. All authors contributed to the article and approved the submitted version.
