# Supplementary material for: A quantitative review of the effects of Se application on the reduction of Hg concentration in plant: a meta-analysis
Source: Front Plant Sci. 2023 Jun 20;14:1199721. doi: 10.3389/fpls.2023.1199721 (PMC10318138; doi:10.3389/fpls.2023.1199721)
Supplement: Supplementary file 1 [file Table_1.docx]

**Supporting Information: Notes S1, Tables S1–S2**

**Notes S1** A list of 38 papers from which the data were extracted for this meta-analysis.

1. He, Y., Yuan, L.X., Tang, J.F., Yin, X.B. (2017). Distribution characteristics of selenium and mercury in seeding rice under selenate fortification (In Chinese). *J. Anhui Agric. Univ.* 44, 726-731. doi: 10.13610/j.cnki.1672-352x.20170811.008
2. Tran, T.A.T., Dinh, Q.T., Cui, Z.W., Huang, J., Wang, D., Wei, T.J., et al. (2018a). Comparing the influence of selenite (Se^4+^) and selenate (Se^6+^) on the inhibition of the mercury (Hg) phytotoxicity to pak choi. *Ecotoxicol. Environ. Saf*. 147, 897-904. doi: 10.1016/j.ecoenv.2017.09.061
3. Tran, T.A.T., Zhou, F., Yang, W.X., Wang, M.K., Dinh, Q.T., Wang, D, et al. (2018b). Detoxification of mercury in soil by selenite and related mechanisms. *Ecotoxicol. Environ. Saf*. 159, 77-84. doi: 10.1016/j.ecoenv.2018.04.029
4. Afton, S.E., Caruso, J.A. (2009). The effect of Se antagonism on the metabolic fate of Hg in *Allium fistulosum*. *J. Anal. At. Spectrom*. 24, 759-766. doi: 10.1039/b823251b
5. Shanker, K., Mishra, S., Srivastava, S., Srivastava, R., Daas, S., Prakash S., et al. (1996). Effect of selenite and selenate on plant uptake and translocation of mercury by tomato (*Lycopersicum esculentum*). *Plant Soil*. 183, 233-238. doi: 10.1007/bf00011438
6. Shanker, K., Mishra, S., Srivastava, R., Srivastava, S., Daas, S., Prakash S., et al. (1996). Study of mercury-selenium (Hg-Se) interactions and their impact on Hg uptake by the radish (*Raphanus sativus*) plant. *Food Chem. Toxicol*. 34, 883-886. doi: 10.1016/s0278-6915(96)00047-6
7. Zhao, J.T., Gao, Y.X., Li, Y.F., Hu, L., Peng, X.M., Dong, Y.X., et al. (2013). Selenium inhibits the phytotoxicity of mercury in garlic (*Allium sativum*). *Environ Res*. 125, 75-81. doi: 10.1016/j.envres.2013.01.010
8. Zhao, J.T., Li, Y.F., Li, Y.Y., Gao, Y.X., Li, B., Hu, Y., et al. (2014). Selenium modulates mercury uptake and distribution in rice (*Oryza sativa* L.), in correlation with mercury species and exposure level. *Metallomics*. 6, 1951-1957. doi: 10.1039/c4mt00170b
9. Li, Y.F., Zhao, J.T., Li, Y.Y., Li, H.J., Zhang, J.F., Li, B., et al. (2015). The concentration of selenium matters: a field study on mercury accumulation in rice by selenite treatment in qingzhen, Guizhou, China. *Plant Soil*. 391, 195-205. doi: 10.1007/s11104-015-2418-4
10. Wang, X., Tam, N.F.Y., Fu, S., Ametkhan, A., Ouyang Y., and Ye, Z.H. (2014). Selenium addition alters mercury uptake, bioavailability in the rhizosphere and root anatomy of rice (*Oryza sativa*). *Ann. Bot*. 114, 271-278. doi: 10.1093/aob/mcu117
11. Zhou, X.B., Yu, S.H., Wang, W.H., Chang, H., Zhou, Y.X. (2014). Effects of application of selenium in soil on the formation of root surface iron plaque and mercury uptake by rice plants (In Chinese). *J. Southwest. Univ. (Natural Science Edition)*. 39, 50-56. doi: 10.13718/j.cnki.xdzk.2014.01.015
12. Pacheco, P., Hanley, T., Figueroa, J.A.L. (2014). Identification of proteins involved in Hg-Se antagonism in water hyacinth (*Eichhornia crassipes*). *Metallomics*. 6, 560-571. doi:
13. Li, Y.Y., Hu, W.J., Zhao, J.T., Chen Q.M., Wang, W., Li, B., et al. (2019). Selenium decreases methylmercury and increases nutritional elements in rice growing in mercury-contaminated farmland. *Ecotoxicol. Environ. Saf*. 182, 109447. doi: 10.1016/j.ecoenv.2019.109447
14. Wang, Y.J., Wei, Z.B., Zeng, Q.L., Zhong, H. (2016a). Amendment of sulfate with Se into soils further reduces methylmercury accumulation in rice. *J. Soils Sediments*. 16, 2720-2727. doi: 10.1007/s11368-016-1453-y
15. Wang, Y.J., Dang, F., Evans, R.D., Zhong, H., Zhao, J.T., and Zhou, D.M. (2016b). Mechanistic understanding of MeHg-Se antagonism in soil-rice systems: the key role of antagonism in soil. *Sci. Rep*. 6, 19477. doi: 10.1038/srep19477
16. Xu, X.H., Yan, M., Liang, L.C., Lu, Q.H., Han, J.L., Liu, L., et al. (2019). Impacts of selenium supplementation on soil mercury speciation, and inorganic mercury and methylmercury uptake in rice (*Oryza sativa* L.). *Environ. Pollut*. 249, 647-654. doi: 10.1016/j.envpol.2019.03.095
17. Tang, W., Dang, F., Evans, D., Zhong, H., Xiao, L. (2017). Understanding reduced inorganic mercury accumulation in rice following selenium application: Selenium application routes, speciation and doses. *Chemosphere*. 169, 369-376. doi: 10.1016/j.chemosphere.2016.11.087
18. Lv, W.Q., Zhan, T.L., Abdelhafiz, M.A., Feng, X.B., Meng, B. (2021). Selenium-amended biochar mitigates inorganic mercury and methylmercury accumulation in rice (*Oryza sativa* L.). *Environ. Pollut*. 291, 118259. doi: 10.1016/j.envpol.2021.118259
19. Wang, Y.J., Dang, F., Zheng, X.M., Zhong, H. (2019). Biochar amendment to further reduce methylmercury accumulation in rice grown in selenium-amended paddy soil. *J. Hazard. Mater.* 365, 590-596. doi: 10.1016/j.jhazmat.2018.11.052
20. Yan, M., Guo, J.Y., Feng, X.B., Zhang, C., Qiu, G.L. (2015). Effects of selenium on mercury speciation and its bioavailability in paddy soils (In Chinese). *Chin. J. Ecol.* 34, 1402-1406. doi: 10.13292/j.1000-4890.20150311.040
21. Zhou, X.B., Wang, W.H., Yu, S.H., Zhou, Y.X. (2013). Interactive effects of selenium and mercury on their uptake by rice seedlings. *Res. J. Appl. Sci. Eng. Technol*. 5, 4733-4739. doi: 10.19026/rjaset.5.4311
22. Li, Y.Y., Zhao, J.T., Gao, Y.X., Li, Y.F., Li, B., Zhao, Y.L. (2014). Effects of iron plaque and selenium on the absorption and translocation of inorganic mercury and methylmercury in rice (*Oryza sativa* L.) (In Chinese). *Asian. J. Ecotoxicol.* 9, 972-977. doi: 10.7524/aje.1673-5897-20140519002
23. Zhou, X.B., Li, Y.Y. (2019). Effect of iron plaque and selenium on mercury uptake and translocation in rice seedlings grown in solution culture. *Environ. Sci. Pollut. Res*. 26, 13795-13803. doi: 10.1007/s11356-018-3066-z
24. Liu, M.Y., Sun, Y.F., Zhou, L.Y., Zheng, X.M., Wang, Y.J. (2022). Effects of the Co-applications of Selenium and Biochar on Methylmercury Accumulation in Rice (In Chinese). *Earth Environ.* 50, 340-345. doi: 10.14050/j.cnki.1672-9250.2022.50.062
25. Chen, S.S. (2009). The study of interrelation between Se and Hg, Cd in the system of soil-plant. Master Dissertation, Guizhou University (In Chinese).
26. Wang, Y. (2015). Effects of selenium on absorption, transportation, and transformation of methylmercury in rice plants. Master Dissertation, Guizhou University (In Chinese).
27. Teng, S.R. (2018). Effects of exogenous se on growth and internal quality of polygonatum sibiricum. Master Dissertation, Hubei University for Nationalities (In Chinese).
28. Guo, C.C. (2019). Effect of selenium addition on mercury activity in soil under low temperature pyrolysis. Master Dissertation, Guizhou Normal University (In Chinese).
29. Chen, L.Y, Wang, G.B., Li, S.X., Wang, Z.B. (2016). Effects of selenium on alfalfa growth, nutrient absorption and nitrogen fixing ability under Pb and Hg stress (In Chinese). *Chin. J. Grassl.* 38, 109-114. doi: 10.16742/j.zgcdxb.2016-05-18
30. Du, S.H, Yu, Z.J. (1987). Interaction between mercury and selenium in plants (In Chinese). *Environ. Sci.* 8, 43-46+77. doi: 10.13227/j.hjkx.1987.06.011
31. Gao, A.X., Zhou, X.B., Zhang, C.M. (2017). Effect of iron plaque on root on uptake and translocation of mercury in rice seedlings treated with selenium (Ⅳ) (In Chinese). *Acta Pedologica Sinica.* 54, 989-998. doi: 10.11766/trxb201610060383
32. Yang, X.H., Gao, Y.X., Wu, G. (2009). Effect of selenium on mercury accumulation in hydroponic garlic (In Chinese). *Environ. Health.* 26, 161-162. doi: 10.16241/j.cnki.1001-5914.2009.02.007
33. Zhang, L. (2016). Interaction mechanism of selenium and mercury in soil-rice system. Master Dissertation, Southwest University. (In Chinese)
34. Dun, C.Y. (2016). The influence on selenium, heavy metal and main bioactive component content and growth of selenium under different concentrations of *Gynostemma pentahyllum* and *Ampelopsis grossedentata*. Master Dissertation, Hubei University for Nationalities (In Chinese).
35. Jiang, N., Yang, C., Wan, Z.X., Chen, G.H., Zheng, X.J. (2016). Sodium selenite regulates growth and uptake of Se, As and heavy metals of *Trillium tschonoskii* (In Chinese). *J. Chin. Med. Mater.* 39, 1960-1965. doi: 10.13863/j.issn1001-4454.2016.09.009
36. Cong, L.Q., Zhang, C.Y., Yang, C. (2012). Influence on selenium application to the uptake of Cd, Cr, Hg and Pb in Chinese medicinal herb *reineckea carnea* (In Chinese). *Guangdong Agric. Sci.* 19, 23-26. doi: 10.3969/j.issn.1004-874X.2012.19.008
37. Zhao, J.T., Liang, X.J., Zhu, N.L., Wang, L.M., Li, Y.Y., Li, Y.F., et al. (2020). Immobilization of mercury by nano-elemental selenium and the underlying mechanisms in hydroponic-cultured garlic plant. *Environ. Sci. Nano*. 7, 1115-1125. doi: 10.1039/c9en01294j
38. Zhang, L., Zhou, X.B., Su, T.T. (2017). Effects of foliar application of selenium on cadmium and mercury absorption in different growth periods of rice (In Chinese). *J. Southwest Univ. (Natural Science Edition).* 39, 50-56. doi: 10.13718/j.cnki.xdzk.2017.07.008

**Table S1** General information for the overall date used in the meta-analysis.

| No. | Type | Species | *k* | Se | c(Se_add_)  (μmol/kg) | c(Se_growth media_)  (μmol /kg) | Hg | c(Hg_plant_)  (μmol /kg) | c(Hg_growth media_)  (μmol /kg) | Se/Hg | References |
| --- | --- | --- | --- | --- | --- | --- | --- | --- | --- | --- | --- |
| 1 | P | Rice | 12 | Ⅵ | 12.66-63.32 | 15.45-66.11 | THg | 0.03-2.24 | 0.16-5.14 | 3.00-421.78 | He et al., 2017 |
| 2 | P | Pak Choi | 24 | Ⅵ | 6.33-31.66 | 8.95-34.28 | THg | 0.37-177.26 | 0.25-15.21 | 0.59-137.54 | Tran et al., 2018a |
| 3 | P | Pak Choi | 24 | Ⅳ | 6.33-31.66 | 8.95-34.28 | THg | 0.28-173.55 | 0.25-15.21 | 0.59-137.54 | Tran et al., 2018b |
| 4 | S | *Allium fistulosum* | 2 | Ⅳ | 379.94 | 379.94 | THg | 0.52-125.85 | 74.78 | 5.08 | Afton & Caruso, 2009 |
| 5 | P, S | Tomato | 120 | Ⅳ, Ⅵ | 6.33-75.99 | 6.33-75.99 | THg | 0.01-2.40 | 9.97-24.93 | 0.25-7.62 | Shanker et al., 1996a |
| 6 | P, S | Radish | 80 | Ⅳ, Ⅵ | 6.33-75.99 | 6.33-75.99 | THg | 0.08-14.35 | 9.97-24.93 | 0.25-7.62 | Shanker et al., 1996b |
| 7 | H | Gallic | 90 | Ⅳ, Ⅵ | 0.13-1266.46 | 0.13-1266.46 | THg | 0.00-5.32 | 0.05-498.53 | 0.00-25404.00 | Zhao et al., 2013 |
| 8 | P, H | Rice | 53 | Ⅳ | 1.27-126.65 | 1.27-126.65 | THg, MeHg | 0.16-480.40 | 0.00-49.85 | 0.03-2540.40 | Zhao et al., 2014 |
| 9 | F | Rice | 5 | Ⅳ | 0.13-63.32 | 8.87-72.06 | THg | 0.19-0.47 | 1176.53 | 0.00-0.06 | Li et al., 2015 |
| 10 | P, H | Rice | 16 | Ⅳ | 1.00-126.65 | 1.00-72.19 | THg, MeHg | 0.03-10265.72 | 0.23-5.00 | 0.20-314.79 | Wang et al., 2014 |
| 11 | P | Rice | 8 | Ⅳ | 12.66-101.32 | 15.64-104.29 | THg | 0.93-10.68 | 10.23 | 1.53-10.19 | Zhou et al., 2014 |
| 12 | H | Water hyacinth | 3 | Ⅳ | 1.27 | 1.27 | THg | 162.02-3978.26 | 0.25 | 5.08 | Pacheco et al., 2014 |
| 13 | F | Rice | 40 | Ⅳ | 0.13-63.32 | 8.87-72.06 | THg, MeHg | 0.12-84.13 | 1179.67 | 0.01-0.06 | Li et al., 2019 |
| 14 | P | Rice | 8 | Ⅳ, Ⅵ | 37.99 | 45.39 | MeHg | 0.08-1.42 | 10.97 | 4.50 | Wang et al., 2016a |
| 15 | P, Fo | Rice | 54 | Ⅳ, Ⅵ | 6.33-75.99 | 17.86-158.94 | MeHg | 0.02-2.33 | 11.72-0.01 | 0.68-207.14 | Wang et al., 2016b |
| 16 | F | Rice | 48 | Ⅳ | 253.29-6332.32 | 256.71-6335.74 | THg, MeHg | 0.03-8.02 | 16.98 | 15.12-373.05 | Xu et al., 2019 |
| 17 | P, Fo | Rice | 36 | Ⅳ, Ⅵ | 6.33-75.99 | - | IHg | 0.18-13.10 | 11.96 | - | Tang et al, 2017 |
| 18 | P | Rice | 4 | Ⅳ | 126.65-633.23 | - | MeHg, IHg | 0.01-0.16 | - | - | Lv et al, 2021 |
| 19 | P | Rice | 6 | Ⅳ, Ⅵ | 37.99-75.99 | - | MeHg | 0.20-1.88 | - | - | Wang et al., 2018 |
| 20 | F | Rice | 6 | Ⅳ | 253.29-6332.32 | - | THg | 0.19-0.61 | - | - | Yan et al., 2015 |
| 21 | H | Rice | 8 | Ⅳ | 184.90-1266.46 | 0.18-1.27 | THg | 0.84-984.86 | 0.50 | 0.37-2.54 | Zhou et al., 2012 |
| 22 | H | Rice | 2 | Ⅳ | 2.50 | 2.50 | THg | 16.62-1533.94 | 2.50 | 1.00 | Li et al., 2014 |
| 23 | H | Rice | 2 | Ⅵ | 2.50 | 2.50 | THg | 17.79-1499.20 | 2.50 | 1.00 | Zhou & Li, 2019 |
| 24 | P | Rice | 18 | Ⅳ, Ⅵ | 37.99-75.99 | 42.43-77.25 | THg, MeHg | 0.02-0.48 | 12.46 | 3.40-6.20 | Liu et al., 2022 |
| 25 | P | Wheat, Sorghum | 162 | Ⅳ | 12.66-37.99 | 27.28-65.27 | THg | 0.01-1.17 | 0.73-15.69 | 1.74-89.07 | Chen, 2009 |
| 26 | H | Rice | 50 | Ⅳ, Ⅵ | 0.15-0.98 | 0.15-0.98 | MeHg | 0.00-0.07 | 0.10 | 1.50-9.80 | Wang, 2015 |
| 27 | S | *Polygonatum sibiricum* | 26 | Ⅳ | 31.66-1053.08 | 31.66-1053.08 | THg | 4.27-23.53 | 74.78 | 0.42-21.17 | Teng, 2018 |
| 28 | P | Rice, Pak Choi | 56 | Ⅳ | 1.27-126.65 | - | Hg | 0.25-227.53 | - | - | Guo, 2019 |
| 29 | H | Alfalfa | 2 | Ⅳ | 63.32 | 63.32 | THg | 0.39-0.44 | 0.00-7.48 | 8.47 | Chen, 2016 |
| 30 | H | Maize | 2 | Ⅳ | 379.94 | 379.94 | THg | 63.74-84.04 | 249.27 | 1.52 | Du & Yu, 1987 |
| 31 | H | Rice | 2 | Ⅳ | 6.33 | 6.33 | THg | 12.45-1752.89 | 1.50 | 4.23 | Gao et al., 2017 |
| 32 | H | Gallic | 3 | Ⅳ | 126.65 | 126.65 | THg | 25.92-3399.32 | 49.85 | 2.54 | Yang et al., 2009 |
| 33 | P, H | Rice | 87 | Ⅳ, Ⅵ | 2.00-101.32 | 2.00-104.29 | THg | 0.95-513.33 | 0.00-10.23 | 0.20-10.19 | Zhang, 2016 |
| 34 | S | *Gynostemma pentahyllum*, *Ampelopsis grossedentata* | 19 | Ⅳ | 6.33-1266.46 | 6.33-1266.46 | THg | 0.26-13.31 | 24.93 | 0.25-50.81 | Dun, 2016 |
| 35 | S | *Trillium tschonoskii* | 7 | Ⅳ | 63.32-506.59 | 63.32-506.59 | THg | 58.95-178.80 | 49.85 | 1.27-10.16 | Jiang et al., 2016 |
| 36 | P | *Reineckea carnea* | 4 | Ⅳ | 50.66-151.98 | - | THg | 0.96-1.32 | 0.73 | - | Cong et al., 2012 |
| 37 | H | Gallic | 90 | Ⅳ, Ⅵ | 0.13-633.23 | 0.13-633.23 | THg | 0.00-9.56 | 0.05-249.26 | 0.00-12702.00 | Zhao et al., 2020 |
| 38 | Fo | Rice | 12 | Ⅳ | 2.53-63.32 | 5.51-66.30 | THg | 1.84-8.54 | 10.23 | 0.54-6.48 | Zhang et al., 2017 |

# P - Pot culture experiment; H - Hydroponic culture experiment; S - Sand culture experiment; F - Field culture experiment; Fo - Foliar fertilization experiment.

**Table S2** General information for the categorical data used in the meta-analysis.

| Variable | | *k* | Articles | Plant  species | Range(Se_add_)  (μmol/kg) | Average Concentration(Se_add_)  (μmol/kg) | Range(Hg_growth media_)  (μmol/kg) | Average Concentration(Hg_growth media_)  (μmol/kg) |
| --- | --- | --- | --- | --- | --- | --- | --- | --- |
| Complete dataset | | 1193 | 38 | 16 | 0.13-6332.32 | 181.87 | 0.00-1179.67 | 69.55 |
| Cultivation method | Pot | 576 | 18 | 7 | 1.27-633.23 | 36.71 | 0.16-207.14 | 17.91 |
|  | Hydroponic | 318 | 13 | 5 | 0.13-1266.46 | 132.15 | 0.00-598.53 | 75.61 |
|  | Sand | 156 | 7 | 8 | 6.33-1583.08 | 196.50 | 0.00-74.78 | 29.93 |
|  | Field | 99 | 4 | 1 | 0.13-6332.32 | 1181.97 | 16.98-1179.67 | 414.95 |
|  | Foliar | 44 | 3 | 1 | 2.53-63.32 | 26.17 | 10.23 | 10.23 |
| Exogenous Se speciation | Se(Ⅳ) | 786 | 32 | 16 | 0.13-6332.32 | 103.63 | 0.00-1179.67 | 81.52 |
|  | Se(Ⅵ) | 401 | 14 | 5 | 0.13-6332.32 | 340.31 | 0.05-498.53 | 47.55 |
| Plant Hg speciation | THg | 959 | 32 | 16 | 0.13-6332.32 | 160.16 | 0.00-1179.67 | 76.94 |
|  | MeHg | 196 | 10 | 1 | 0.13-6332.32 | 310.35 | 0.10-207.14 | 34.24 |
|  | IHg | 38 | 2 | 1 | 6.33-633.23 | 77.14 | 11.96 | 11.96 |
| Se/Hg molar ratio | Se/Hg ≤1 | 187 | 18 | 7 | 0.13-126.65 | 15.95 | 0.50-1179.67 | 282.01 |
|  | 1< Se/Hg ≤3 | 216 | 20 | 11 | 0.13-1266.46 | 75.90 | 0.05-498.53 | 34.83 |
|  | Se/Hg >3 | 509 | 25 | 14 | 0.50-6332.32 | 331.74 | 0.05-74.78 | 11.09 |
| Plant Hg BAF | Root | 329 | 24 | 10 | 0.13-6332.32 | 133.16 | 0.05-1179.67 | 57.59 |
|  | Stem | 70 | 6 | 3 | 0.13-6332.32 | 356.70 | 0.05-498.53 | 110.73 |
|  | Leaf | 89 | 7 | 5 | 0.13-6332.32 | 373.24 | 0.05-498.53 | 92.41 |
|  | Grain | 82 | 10 | 3 | 0.13-6332.32 | 186.33 | 0.05-1179.67 | 181.09 |
| Growth stage | Seedling | 195 | 12 | 5 | 0.15-379.94 | 22.73 | 0.00-249.26 | 8.88 |
|  | Mature | 411 | 17 | 4 | 0.13-6332.32 | 339.55 | 0.23-1179.67 | 132.97 |
| Rice only | | 517 | 22 | 1 | 0.13-6332.32 | 269.20 | 0.00-1179.67 | 92.98 |
| Cultivation method | Pot | 244 | 12 | 1 | 1.27-633.23 | 47.60 | 0.16-207.14 | 30.03 |
|  | Hydroponic | 130 | 8 | 1 | 0.15-126.65 | 12.47 | 0.00-48.85 | 5.06 |
|  | Field | 99 | 4 | 1 | 0.13-6332.32 | 1181.97 | 16.98-1179.67 | 414.95 |
|  | Foliar | 44 | 3 | 1 | 2.53-63.32 | 26.17 | 10.23 | 10.23 |
| Exogenous Se speciation | Se(Ⅳ) | 324 | 17 | 1 | 0.13-6332.32 | 73.57 | 0.00-1179.67 | 149.33 |
|  | Se(Ⅵ) | 187 | 9 | 1 | 0.15-6332.32 | 629.02 | 0.10-207.14 | 20.49 |
| Plant Hg speciation | THg | 283 | 16 | 1 | 0.13-6332.32 | 257.97 | 0.00-1179.67 | 133.03 |
|  | MeHg | 196 | 10 | 1 | 0.13-6332.32 | 310.35 | 0.10-207.14 | 34.24 |
|  | IHg | 38 | 2 | 1 | 6.33-633.23 | 77.14 | 11.96 | 11.96 |
| Se/Hg molar ratio | Se/Hg ≤1 | 71 | 10 | 1 | 0.13-63.32 | 10.76 | 0.50-1179.67 | 473.53 |
|  | 1< Se/Hg ≤3 | 82 | 8 | 1 | 0.15-126.65 | 14.88 | 0.10-49.85 | 8.68 |
|  | Se/Hg >3 | 206 | 12 | 1 | 0.50-126.65 | 535.64 | 0.05-16.98 | 8.67 |
| Plant Hg BAF | Root | 118 | 13 | 1 | 0.13-6332.32 | 131.11 | 0.05-1179.67 | 77.00 |
|  | Stem | 8 | 2 | 1 | 12.66-6332.32 | 1628.99 | 10.23-16.98 | 15.30 |
|  | Leaf | 8 | 2 | 1 | 12.66-6332.32 | 1628.99 | 10.23-16.98 | 15.30 |
|  | Grain | 64 | 9 | 1 | 0.13-6332.32 | 232.20 | 0.23-1179.67 | 229.42 |
| Growth stage | Seedling | 142 | 9 | 1 | 0.15-126.65 | 14.63 | 0.00-49.85 | 4.86 |
|  | Elongation | 19 | 2 | 1 | 2.53-101.32 | 44.13 | 10.23 | 10.23 |
|  | Booting | 27 | 2 | 1 | 2.53-101.32 | 45.12 | 10.23 | 10.23 |
|  | Mature | 329 | 16 | 1 | 0.13-6332.32 | 425.69 | 0.23-1179.67 | 173.38 |
| Plant part | Root | 161 | 18 | 1 | 0.13-6332.32 | 192.88 | 0.00-1179.67 | 61.53 |
|  | Stem | 33 | 5 | 1 | 1.27-6332.32 | 916.96 | 10.23-16.98 | 14.95 |
|  | Leaf | 22 | 3 | 1 | 1.27-6332.32 | 1192.89 | 10.23-16.98 | 16.02 |
|  | Grain | 134 | 16 | 1 | 0.13-6332.32 | 370.84 | 0.23-1179.67 | 172.25 |
| Rice excluded | | 676 | 17 | 15 | 0.13-1583.08 | 119.21 | 0.00-498.53 | 53.19 |
| Cultivation method | Pot | 332 | 7 | 6 | 1.27-151.98 | 28.70 | 0.25-15.69 | 8.03 |
|  | Hydroponic | 188 | 5 | 4 | 0.13-1266.46 | 214.91 | 0.05-498.53 | 124.39 |
|  | Sand | 156 | 7 | 8 | 6.33-1583.08 | 196.50 | 0.00-74.78 | 29.93 |
| Exogenous Se speciation | Se(Ⅳ) | 462 | 16 | 14 | 0.13-1583.08 | 123.66 | 0.00-498.53 | 43.85 |
|  | Se(Ⅵ) | 214 | 5 | 4 | 0.13-1266.46 | 109.60 | 0.05-498.53 | 75.77 |
| Se/Hg molar ratio | Se/Hg ≤ 1 | 116 | 8 | 6 | 0.13-126.65 | 19.12 | 4.99-498.53 | 164.79 |
|  | 1 < Se/Hg ≤ 3 | 134 | 12 | 10 | 0.13-1266.46 | 113.24 | 0.05-498.53 | 50.83 |
|  | Se/Hg > 3 | 339 | 13 | 13 | 1.27-1583.08 | 193.13 | 0.05-74.78 | 12.73 |
| Plant Hg BAF | Root | 211 | 11 | 9 | 0.13-1583.08 | 121.60 | 0.05-498.53 | 46.63 |
|  | Stem | 62 | 4 | 2 | 0.13-1266.46 | 192.53 | 0.05-498.53 | 123.04 |
|  | Leaf | 81 | 5 | 4 | 0.13-1266.46 | 249.22 | 0.05-498.53 | 100.23 |
|  | Grain | 18 | 1 | 2 | 0.13-37.99 | 23.23 | 0.05-15.69 | 9.24 |
| Growth stage | Seedling | 53 | 3 | 4 | 12.66-379.94 | 44.45 | 0.73-249.26 | 19.66 |
|  | Mature | 82 | 2 | 3 | 1.27-126.65 | 27.55 | 0.73-15.69 | 8.66 |
